# Discovery of malathion resistance QTL in *Drosophila melanogaster* using a bulked phenotyping approach

**DOI:** 10.1093/g3journal/jkac279

**Published:** 2022-10-17

**Authors:** Stuart J Macdonald, Anthony D Long

**Affiliations:** Department of Molecular Biosciences, University of Kansas, Lawrence, KS 66046, USA; Center for Computational Biology, University of Kansas, Lawrence, KS 66047, USA; Department of Ecology and Evolutionary Biology, University of California at Irvine, Irvine, CA 92697, USA

**Keywords:** X-QTL, extreme QTL, MPP, DSPR, malathion, insecticide resistance

## Abstract

*Drosophila melanogaster* has proved an effective system with which to understand the evolutionary genetics and molecular mechanisms of insecticide resistance. Insecticide use has left signatures of selection in the fly genome, and both functional and quantitative genetic studies in the system have identified genes and variants associated with resistance. Here, we use *D. melanogaster* and leverage a bulk phenotyping and pooled sequencing “extreme quantitative trait loci” approach to genetically dissect variation in resistance to malathion, an organophosphate insecticide. We resolve 2 quantitative trait loci, one of which implicates allelic variation at the cytochrome P450 gene *Cyp6g1*, a strong candidate based on previous work. The second shows no overlap with hits from a previous genome-wide association study for malathion resistance, recapitulating other studies showing that different strategies for complex trait dissection in flies can yield apparently different architectures. Notably, we see no genetic signal at the *Ace* gene. *Ace* encodes the target of organophosphate insecticide inhibition, and genome-wide association studies have identified strong *Ace*-linked associations with resistance in flies. The absence of quantitative trait locus implicating *Ace* here is most likely because our mapping population does not segregate for several of the known functional polymorphisms impacting resistance at *Ace*, perhaps because our population is derived from flies collected prior to the widespread use of organophosphate insecticides. Our fundamental approach can be an efficient, powerful strategy to dissect genetic variation in resistance traits. Nonetheless, studies seeking to interrogate contemporary insecticide resistance variation may benefit from deriving mapping populations from more recently collected strains.

## Introduction

Increased agricultural productivity over the last century has been, at least in part, promoted by broader use of insecticides to protect crops against insect pests (Tudi *et al.* 2021). Equally, insecticides are widely employed to protect humans against insect vectors of infectious disease (World Health Organization 2006). A side-effect of this widespread insecticide use is that populations of many insect species—those that are the targets of insecticides, as well as nontarget species—have evolved strong resistance to important classes of insecticides (e.g. Hemingway and Ranson 2000; Rivero *et al.* 2010; Moyes *et al.* 2017).

A route to understanding insecticide resistance mechanisms is to resolve the variants that have been selected in populations (Hawkins *et al.* 2019). Critical insight into the genetic evolution of resistance has come from direct work with a range of pest insect/insect vector systems, including various *Anopheles*/*Culex* mosquito species (see Hemingway *et al.* 2004), and *Lucilia* blowflies (e.g. Hartley *et al.* 2006). Nonetheless, despite not itself being either a disease vector or a crop pest (although see Hall *et al.* 2018) *Drosophila melanogaster* has emerged as a useful and practical system with which to study insecticide resistance (Ffrench-Constant *et al.* 2004; Scott and Buchon 2019). This is due to the relative ease with which *D. melanogaster* can be genetically manipulated to test the molecular genetic role of genes/variants, the availability of a huge array of genome scale data on sequence/transcriptome variation in *D. melanogaster*, and the existence of living resources enabling the genetic dissection of trait variation (King, Macdonald, *et al.* 2012; King, Merkes, *et al.* 2012; Mackay *et al.* 2012; Huang *et al.* 2014). Additionally, studies have demonstrated that natural populations of *D. melanogaster* have been impacted by insecticide use, with signatures of adaptation around genes known to be involved in the response to insecticide exposure (Karasov *et al.* 2010; Garud *et al.* 2015; Duneau *et al.* 2018).

Several studies have sought to use the DGRP (*Drosophila* Genetic Reference Panel), a panel of ∼200 sequenced inbred strains (Mackay *et al.* 2012; Huang *et al.* 2014), to resolve the genetic architecture of insecticide resistance in this lab-derived *D. melanogaster* population using a genome-wide association study (GWAS) design. In some cases, studies have recovered GWAS hits at known insecticide targets, such as the *Ace* (*Acetylcholine esterase*, FBgn0000024) gene (Battlay *et al.* 2018; Duneau *et al.* 2018) which can be inhibited by organophosphate and carbamate insecticides. Additionally, GWAS studies have implicated variation at genes that encode cytochrome P450 detoxification enzymes, such as *Cyp6g1*/FBgn0025454 (Battlay *et al.* 2016, 2018) and *Cyp6a23*/FBgn0033978 (Battlay *et al.* 2018; Duneau *et al.* 2018) in insecticide resistance in flies.

The present study was motivated by 2 observations. First, genetic dissection regimes relying on panels of inbred fly strains can be quite inefficient to execute. It is a technical challenge to accurately measure a phenotype on multiple individuals, from both sexes, from dozens to hundreds of different strains. Additionally, such designs are subject to confounding microenvironmental noise, since different genotypes are reared/assayed in different vials/bottles. Approaches that allow test individuals to be communally reared/assayed and that enable bulked phenotyping can mitigate these issues.

Second, for a given phenotype, the genetic architecture uncovered can depend on the set of genotypes employed and the strategy used for genetic dissection. In addition to the DGRP, a number of studies have used the *Drosophila* Synthetic Population Resource (DSPR; King, Macdonald, *et al.* 2012; King, Merkes, *et al.* 2012) to dissect trait variation. The DSPR consists of 2 sets of genotyped recombinant inbred lines (RILs), each derived from an advanced generation intercross initiated with 8 inbred founder strains, that enable quantitative trait locus (QTL) mapping (Long *et al.* 2014). For 2 virus infection phenotypes, studies in both the DGRP (Magwire *et al.* 2012) and DSPR (Cogni *et al.* 2016) have strongly implicated variation at the same loci [the *pst*/FBgn0035770 gene for resistance to *Drosophila* C Virus infection, and the *ref(2)P*/FBgn0003231 gene for resistance to the sigma virus DMelSV]. However, dissection of 3 xenobiotic and stress resistance phenotypes in both the DSPR and DGRP revealed distinct architectures (Najarro *et al.* 2015, 2017; Everman *et al.* 2019); the DSPR suggesting a significant fraction of trait heritability is encapsulated by a handful of mapped loci, whereas in the DGRP few variants yield hits passing a strict, genomewide threshold, and even subthreshold associations are not enriched within the QTL intervals resolved by the DSPR. Since studies examining variation in insecticide resistance have emphasized the DGRP system (Battlay *et al.* 2016, 2018; Denecke *et al.* 2017; Schmidt *et al.* 2017; Duneau *et al.* 2018; Green *et al.* 2019), an investigation of the genetic architecture of insecticide resistance that derives from other approaches is of interest.

In the present study, we dissect variation in resistance to malathion, an organophosphate insecticide, using an “extreme QTL” or X-QTL mapping design (Ehrenreich *et al.* 2010). An X-QTL strategy involves (1) developing a segregating population, (2) selecting individuals with extreme trait values, and (3) comparing allele frequencies between pools of selected and—otherwise matched—control individuals. Here, we employ bulked phenotyping and pooled sequencing of malathion-selected and control individuals derived from a population constructed by mixing several hundred DSPR RILs. Our use of this design in a previous study of caffeine resistance recapitulated QTL identified via traditional RIL-by-RIL phenotyping (Najarro *et al.* 2015; Macdonald *et al.* 2022), while being—in our opinion—more efficient to execute. Since variation in malathion resistance has been examined previously using the DGRP—Battlay *et al.* (2018) found very strong associations at *Ace* and a more modest association at *Cyp6g1—*we could execute a direct comparison of the outcomes of 2 different approaches to genetic analysis in flies.

## Materials and methods

### Fly population

The X-QTL base population used here is a derivation of the one created by Macdonald *et al.* (2022), and consists of the outbred descendants of a mix of RILs from the DSPR collection (King, Macdonald, *et al.* 2012; King, Merkes, *et al.* 2012).

Briefly, 8 highly inbred strains (King, Merkes, *et al.* 2012; Chakraborty *et al.* 2019) were used to found a synthetic population that was maintained at large population size for 50 generations. Subsequently, a large set of 8-way, advanced generation RILs were developed via 25 generations of sibling mating (yielding the DSPR lines). The X-QTL base population was created by collecting 10 embryos from each of 663 DSPR pA (“panel A”) RILs, and releasing emerged adults into a 1 cubic foot population cage. The population was maintained in this cage for 42 weeks in an incubator (25°C, 50% relative humidity, 12-h light/12-h dark), replacing all 9–12 rearing bottles in the cage approximately every 14 days. Following this period flies were moved to an 8 cubic foot population cage, the cage was moved out of the incubator (so was subject to a more variable environment), and each week 3 of the 12 rearing bottles in the cage were switched for fresh bottles. Aside from the transition from a small to a large cage, the populations—and any waste and dead flies outside of the frequently replaced rearing bottles—remained in the cage throughout population maintenance. Assuming that the base population experienced 1 generation every 2 weeks under the maintenance regime described above, eggs were collected from the population ∼28 (experimental replicate 1) and ∼48 (replicate 2) generations after its founding from DSPR RILs. Adults emerging from these eggs were used for the bulk malathion resistance assay described below.

It is reasonable to ask whether the extended period of maintenance prior to executing the current X-QTL experiment impacted the haplotype frequencies in the mixed base population. We assessed this by comparing frequencies in the control, unselected samples from the present experiment, with those from a previous X-QTL study that used the exact same base population, but which was executed after only 5 generations of maintenance (Macdonald *et al.* 2022). Supplementary Figure 1 suggests there is no marked change in haplotype frequencies between these datasets.

### Rearing experimental animals

For each experimental replicate, test animals were derived from the base population as follows, with additional detail in Macdonald *et al.* (2022). We added five 100-mm diameter petri dishes containing apple juice agar and a small dab of live yeast paste to the base population cage overnight. The next day eggs were removed from plates, suspended in 1× PBS (phosphate-buffered saline), rinsed with additional PBS, and 12-μl of eggs were pipetted into standard *Drosophila* rearing vials (Fisher Scientific, AS515) each containing ∼10-ml of cornmeal–yeast–molasses media (see Supplementary Text 1). Egg pipetting in this fashion has been shown to yield relatively homogeneous egg density over vials (Clancy and Kennington 2001), and in our hands yields ∼70 adults of each sex. Two days following the first emergence of adults, all emerged flies were moved to fresh media vials. The next day, we sexed flies over CO_2_ anesthesia, collecting a series of single-sex groups of 50 flies into fresh media vials. Flies—then 3–5 days old—were assayed the following day. All rearing, maintenance, and testing (below) of experimental flies was conducted at 25°C, 50% relative humidity, and on a 12-h light/12-h dark cycle.

### Collecting control, unselected animals

From each single-sex vial of experimental flies we aspirated 4 flies prior to the malathion resistance assay. These arbitrarily collected control flies represent a sample of the base population allele frequency. We collected a total of 120 control males and females for replicate 1, although only a subset (65 and 43, respectively) were employed for pooled DNA isolation in order to match the number of malathion-selected animals obtained (below). We collected 264 control flies of each sex for replicate 2, all of which were used for pooled DNA isolation.

### Malathion resistance assay

The design of the assay was largely copied from previous studies of insecticide resistance in flies (e.g. Schmidt *et al.* 2010, 2017; Battlay *et al.* 2018). Malathion (Millipore-Sigma, 36143-100MG) was added to acetone (Fisher Chemical, A929) at a concentration of 2 μg/ml, and 500-μl of the mix was added to a series of 20-ml glass scintillation vials (Fisher Scientific, 03-337-5). The interior walls of these vials were coated in malathion by rolling on a hotdog warmer (Grand Slam, HDRG12) with the heat off for ∼15-min in a fume hood to evaporate the acetone. Malathion-coated scintillation vials were left overnight at room temperature before being used for the assay.

Test flies were tipped from single-sex holding vials to malathion-containing vials, and these were plugged with 1/2 of a large cotton ball (VWR, 14224-518) dampened with 1-ml of 10% sucrose solution (Fisher Chemical, S5). Assays were initiated within the first (replicate 1) or second (replicate 2) hour following lights on. After a period of exposure (210–245 min for replicate 1, 190–230 min for replicate 2), flies—both living and dead—were transferred from malathion vials to normal media vials and left for 24 h. This was done because pilot experiments indicated that a fraction of the animals remaining alive immediately following the assay would succumb to the effects of the insecticide after several hours. Plus, discriminating live from dead animals was considerably easier after this period.

### Collecting malathion-selected animals

One day following the assay, alive and dead animals were separated over CO_2_ and counted. In replicate 1, 65 of 1,303 males (5.0%) and 43 of 1,338 females (3.2%) survived, while in replicate 2, 403 of 2,881 males (14.0%) and 693 of 2,817 females (24.6%) survived. Given simulation data presented in Macdonald *et al.* (2022), the scale of the experiment was lower than optimal in replicate 1, while the intensity of the selection was suboptimal for replicate 2.

### DNA isolation, library construction, and sequencing

We isolated DNA from each pool of animals (2 replicates × 2 treatments × 2 sexes = 8 total pools) via the Gentra Puregene Cell Kit (Qiagen, 158767) using straightforward extensions of the manufacturer’s protocol, and the resulting DNA was quantified using a fluorometer (Qubit dsDNA BR Assay Kit, ThermoFisher, Q32853). Subsequently, we used 400-ng of each DNA sample to construct indexed sequencing libraries (Illumina DNA Prep Tagmentation, 20018705; Illumina Nextera DNA CD Indexes, 20018708). Libraries were mixed by replicate into two 4-plexes, and sequenced on an Illumina NextSeq 550 instrument. We obtained 21.2–26.0 million PE150 read pairs for each replicate 1 sample, and 31.2–37.7 million PE75 for each replicate 2 sample.

### Read mapping, SNP calling, and haplotype frequency estimation

For each of the 8 X-QTL pooled sequencing samples, along with the set of 8 inbred strains that founded the pA DSPR population (King, Merkes, *et al.* 2012), raw reads were first mapped to the *D. melanogaster* reference genome (Release 6, dm6) via bwa-mem (Li 2013). This resulted in 50× coverage for the replicate 1 female pools, and 32–39× coverage for the remaining pools. Previous analytical and experimental work indicates coverage at this level enables robust haplotype frequency estimation with the DSPR-based X-QTL design (Macdonald *et al.* 2022). Next, the bcftools mpileup, call and query commands (Li 2011) were employed to generate a file of REF and ALT counts at all SNPs for each sample (founders plus X-QTL samples), and this was converted to REF allele frequencies per sample per SNP (typically yielding REF frequencies of 0 or 1 for the inbred founders).

We call haplotypes for each X-QTL sample in windows of 1.5 cM, stepping through the genome in 0.05-cM increments, using a procedure described in more detail previously (Linder *et al.* 2020; Macdonald *et al.* 2022). Briefly, for each X-QTL pooled sample, and within each window, we use the R/limSolve package (Soetaert *et al.* 2009) to find the set of 8 proportions (summing to 1) that minimizes the sum of the weighted squared differences between the known founder haplotypes, and the observed frequency of each SNP in the window in that sample. Experimental validation of the accuracy of this approach in an 18-way yeast population, and an 8-way DSPR-derived population is presented elsewhere (Linder *et al.* 2020; Macdonald *et al.* 2022).

### X-QTL genome scan

Mapping is performed by executing a statistical test at each window (above) along the genome. First, the set of 8 inferred founder haplotype (*H*) frequencies from each replicate (*R*), and from the control and malathion-selected treatments (*T*) are arcsine square-root transformed (*ASF*). We chose to treat the male and female tests within each experimental replicate as independent, so *R *=* *4 (2 experimental replicates × 2 sexes), and we are therefore geared to identify effects that are consistent in each sex. We then test for differentiation between treatments using the ANOVA *ASF* ∼ *H* + *TRT* + *H*×*TRT*, testing for the effect of the *H*×*TRT* interaction using *R *×* H*×*TRT* as the error term, returning −log_10_(*P*) values. Previous simulation work over a broad parameter space indicates that −log_10_(*P*) = 4 holds the QTL false positive rate roughly at ∼5% genomewide (Linder *et al.* 2020; Macdonald *et al.* 2022). However, we acknowledge the present study is at the lower end of the factors that impact power of the DSPR-based X-QTL design (Macdonald *et al.* 2022). Finally, following smoothing of the −log_10_(*P*) values across each chromosome (via LOESS, to accommodate window-to-window variation in the test statistic), we called QTL peaks, and automatically extracted 3 −log_10_(*P*) drop (“LOD drop”) confidence intervals, which in simulations encapsulate the true position of the causative locus ∼95% of the time (Macdonald *et al.* 2022).

### Gene functional annotations

We used FlyBase (version FB2022_03, Gramates *et al.* 2022) to identify plausible candidate genes within mapped QTL, marking genes if they were tagged with the Gene Ontology terms “detoxification” (GO: 0098754) or “response to insecticide” (GO: 0017085), or if they are members of the following FlyBase Gene Groups—all known players in the detoxification pathway (Xu *et al.* 2005); cytochrome P450 genes (FBgg0001222), glutathione s-transferases (FBgg0000077), other carboxylesterases (FBgg0001375), GT1 family of UDP-glycosyltransferases (FBgg0000797), or ATP-binding cassette transporters (FBgg0000547).

### Testing for a heritable effect of malathion selection

Successful selection for malathion resistance in a population should result in the progeny of the selected population showing increased resistance. For experimental replicate 1, prior to freezing animals for subsequent DNA isolation, we allowed the control animals (120 males and 120 females) and the malathion-selected animals (65 males and 43 females) to lay eggs in regular media vials for ∼24-h. In the following generation, we transferred mixed-sex groups of progeny adults to fresh vials 2 days following the first adult emergence, left flies to mate/age for 48 h, then collected 20 vials of 10 female progeny from both the control and selected populations over CO_2_ anesthesia. The following day (at around 1-h following lights on), when progeny animals were 3–5 days old, all 40 vials were tipped into malathion exposure vials (described above) and the number of dead flies were manually counted by the same investigator periodically over the next 9–10 h. Nearly all flies died during this period.

## Results and discussion

We sought to employ a bulk phenotyping/genotyping X-QTL strategy to resolve genomic regions contributing to resistance to the insecticide malathion in *D. melanogaster*. The base population for selection was generated by mixing several hundred strains from the DSPR collection (King, Macdonald, *et al.* 2012; King, Merkes, *et al.* 2012), resulting in an outbred, highly recombinant population segregating for at most 8 haplotypes at any given position. Subsequently, our experiment followed the same fundamental design we have employed to map caffeine resistance QTL (Macdonald *et al.* 2022). Over 2 experimental replicates, samples of male and female flies from the base population were exposed to malathion, and after a period of exposure, surviving animals were retained. Each pool of malathion-selected animals, along with matching pools of unselected, control animals sampled randomly from the preexposure cohorts of experimental animals, were subjected to bulk DNA isolation and sequencing library construction (2 replicates × 2 sexes × 2 treatments = 8 X-QTL samples in total). Following sequencing, we inferred the frequencies of the 8 possible haplotypes for each of the pooled X-QTL samples at intervals across the genome. Finally, we executed a test to identify consistent differentiation between control and selected samples at each position, resolving locations—QTL—showing significant allele-frequency shifts.

### Selection results in progeny with greater malathion resistance

**Fig. 1.**
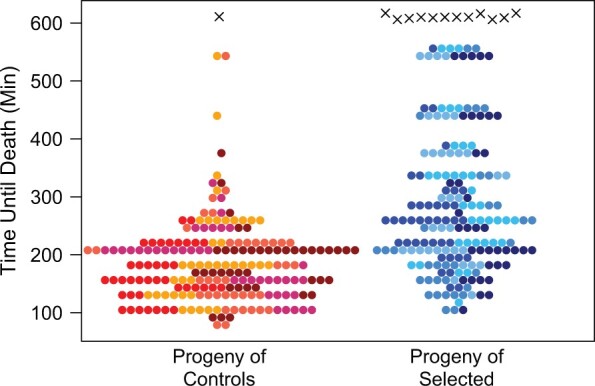
Female progeny of malathion-selected animals is more resistant to the insecticide than progeny of controls. Around 200 female progeny of the replicate 1 cohorts of selected/control animals were assayed for malathion resistance, and the number of dead animals was counted periodically over a ∼10-h exposure period. Each point represents a single female. Animals dying during the exposure period (filled circles) are assumed to have died at the midpoint between sequential counting times. The 5 rearing vials of origin for each sample of test flies are represented by different types of filled circle. Those animals dying after the exposure period (crosses) are presented at the last time they were scored as alive. The progeny of selected females are significantly more resistant to malathion (*t*-test, *P *<* *10^−16^).

Prior to freezing off selected and control animals for experimental replicate 1, the 2 cohorts were allowed to lay eggs, and their adult female progeny tested for malathion resistance. In the ∼10-h over which flies were monitored, 199/200 (99.5%) control female progeny and 183/195 (93.8%) selected female progeny died. Even ignoring these “censored” individuals, progeny of malathion-selected cohorts live substantially longer than progeny of control animals (Fig. 1); the control and selected means are 185 and 286 min, respectively (Welch’s *t*-test = 9.98, *P *<* *10^−16^). This suggests that malathion-selected pools of individuals are enriched for alleles that confer greater resistance to the toxic effects of the insecticide.

### Two mapped autosomal loci contribute to malathion resistance in the DSPR

We considered the male and female tests within each experimental replicate as independent, yielding 4 control-selected pairs of samples. We did this—rather than considering sexes separately—since we are likely underpowered to identify sex-specific effects given our limited level of replication (Macdonald *et al.* 2022). Pooling across sexes is supported by the result that males and females from the same set of 170 DGRP strains have strongly correlated malathion resistance phenotypes (Pearson’s *r* of 0.81–0.89), and by association mapping that appears to implicate the same major malathion resistance loci in both sexes (Battlay *et al.* 2018).

**Fig. 2.**
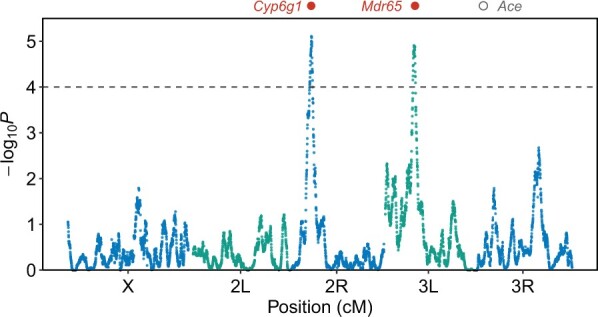
Two QTL for malathion resistance in the DSPR. The −log_10_(*P*) value is the result of contrasting haplotype frequencies of all pairs of control and selected populations in a series of 1.5-cM windows along the genome. Peaks surviving a −log_10_(*P*) = 4 threshold are apparent on chromosome arms 2R and 3L. The locus on 2R includes the well-known *Cyp6g1* insecticide resistance candidate gene, which was previously implicated in natural malathion resistance by a GWAS (Battlay *et al.* 2018). An ABC transporter gene *Mdr65* that impacts insecticide resistance when knocked out/down (Denecke *et al.* 2017; Sun *et al.* 2017) is within the 3L interval. There is no indication that the *Ace* gene—a target of inhibition by organophosphates such as malathion, known to segregate for functional variation impacting insecticide resistance in flies, and a major GWAS hit in a previous study of malathion resistance (Battlay *et al.* 2018)—is associated with phenotype in our DSPR-based X-QTL mapping study; The maximum −log_10_(*P*) score in a 2-Mb window centered on *Ace* is 0.83. Supplementary Figure 2 presents “zoomed in” views of the 2 QTL intervals, highlighting the positions of those genes listed in Table 2.

An X-QTL genome scan contrasting control and selected population frequencies was executed across the genome to identify consistent haplotype frequency shifts due to selection, and analysis revealed 2 peaks rising above a −log_10_(*P*) = 4 threshold, 1 on each autosome (Fig. 2). Confidence intervals on QTL locations implicate fairly wide intervals, and hundreds of protein-coding genes (Table 1), likely due to the limited replication in our experiment (see Macdonald *et al.* 2022).

**Table 1.** Mapped malathion resistance QTL.

Chr	Peak position (bp)^*a*^	Physical interval (bp)^*a*^,^*b*^	QTL size^*b*^		
			Mb^*a*^	**cM**	Genes^*c*^
Chr2R	12,294,089	10,966,645–13,213,848	2.25	4.7	344
Chr3L	6,162,469	5,515,636–6,735,645	1.22	3.8	145

a Based on Release 6 (dm6) of the *D. melanogaster* reference genome.

b Intervals are defined as 3 −log_10_(*P*) drops from the QTL peak.

c Specifically, the number of protein-coding genes in the interval. See Supplementary Table 1 for additional details on these genes.

**Fig. 3.**
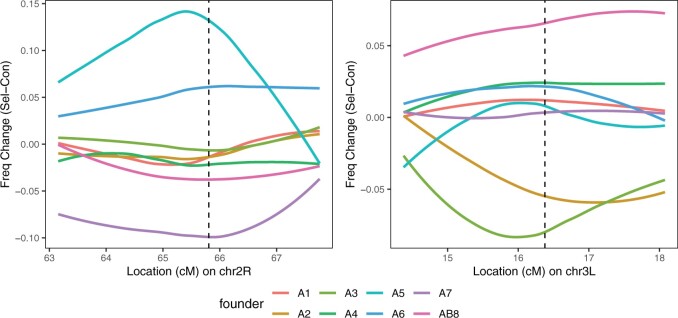
Haplotype frequency differences between control and selected groups at mapped X-QTL. Each plot shows the frequency difference (selected minus control) for each of the 8 founder haplotypes through the 3 −log_10_(*P*) drop interval implicated by each QTL (left = Chr2R, right = Chr3L), with QTL peaks indicated by vertical dashed lines. Values above zero indicate the haplotype frequency is greater in the selected group.

**Fig. 4.**
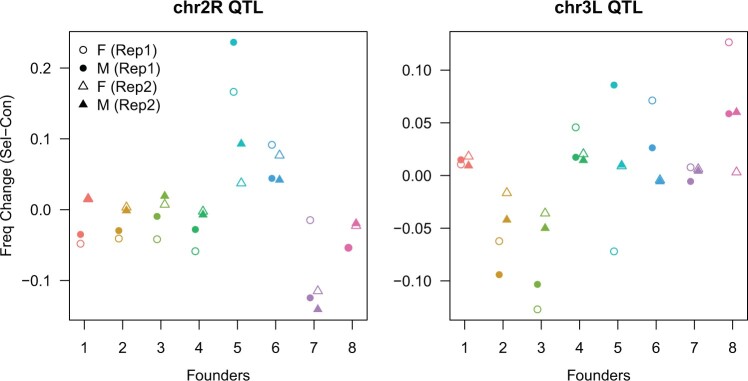
Consistent haplotype frequencies over experimental replicates/sexes at mapped QTL peaks. Each plot shows the frequency difference (selected minus control) for each of the 8 DSPR pA founder haplotypes (1–7 = A1–A7, 8 = AB8) at the 2 X-QTL peaks, separately for each experimental replicate (Rep1, Rep2) and sex (F, females; M, males).

A feature of any mapping system based on a multiparent cross is that the parental haplotypes at a mapped locus can be interrogated for whether they possess phenotype increasing/decreasing alleles. Figure 3 shows that the Chr2R QTL is “driven” by resistance alleles harbored by founders A5 and A6 (these alleles increase in frequency in the selected samples), while A7 appears to confer susceptibility (and the allele decreases in frequency in the selected samples). Similarly, at the Chr3L locus it appears that the AB8 founder allele confers greater resistance, while A2/A3 reduce resistance. Notably, examination of the haplotype frequency differences between each matched pair of control and selected pools (Fig. 4) suggests there is general concordance between frequencies in males and females, suggesting that our analysis of the data without regard to sex was reasonable.

### A well-known insecticide resistance gene, *Cyp6g1*, resides within the Chr2R mapped locus

Both of our X-QTL intervals encompass fairly large numbers of genes (Table 1 and Supplementary Table 1), so we attempted to identify plausible candidate genes by marking those that fall into any of 7 formally defined functional categories relevant to detoxification and insecticide resistance (see *Materials and methods*). We also made use of results from Salces-Ortiz *et al.* (2020, see their Supplementary Table 1) to highlight genes that show differential expression in adult female gut tissue in response to malathion.

For the Chr2R QTL, 9 genes have annotations suggesting a role in detoxification and/or insecticide resistance (Table 2), and 7 of these were shown to be differentially expressed in response to malathion (Salces-Ortiz *et al.* 2020). Cytochrome P450 gene *Cyp6g1* emerges as the strongest candidate to underlie variation at this locus. The gene has a well-defined role in resistance to DDT and other insecticides (Daborn *et al.* 2001, 2002; Chung *et al.* 2007; Schmidt *et al.* 2010; Harrop *et al.* 2014), population-based GWAS have identified hits in/near *Cyp6g1* for malathion (Battlay *et al.* 2018) and azinphos-methyl resistance (Battlay *et al.* 2016), and a *Cyp6g1* knockout reduces resistance to malathion (Battlay *et al.* 2018). Additionally, A6—one of the 2 founders conferring resistance at this QTL (Fig. 3)—possesses 2 copies of *Cyp6g1*, 1 with a full-length, and 1 with a fragment of an *Accord* transposable element (TE) just upstream of the start of the gene copy (Chakraborty *et al.* 2019; DSPR Variant UCSC Browser 2019). Previous work has indicated an *Accord* insertion in this region increases *Cyp6g1* transcription (Daborn *et al.* 2002; Chung *et al.* 2007). Indeed, individuals carrying the A6 haplotype at *Cyp6g1* exhibit higher gene expression in adult female heads than those carrying other DSPR founder haplotypes at this position (King *et al.* 2014; Chakraborty *et al.* 2019).

**Table 2.** Plausible candidate genes present within QTL intervals.

QTL	Gene symbol^a^	Functional evidence^b^	Malathion DE response^c^
Chr2R	*Cyp12d1-dd*	P450, insecticide	B+/C+
	*Cyp12d1-pd*	P450, insecticide	B+/C+
	*Sod3*	Detox	NS
	*Cyp6g1*	P450, detox, insecticide	B+/D−
	*Cyp6g2*	P450, detox, insecticide	D−
	*Cyp6t3*	P450, detox, insecticide	NS
	*Cyp301a1*	P450	C−/D−
	*Cyp9h1*	P450	C+
	*Mdr49*	ABC, insecticide	B+/D−
Chr3L	*Spo*	P450	D+
	*CG10226*	ABC	B+/C+
	*Mdr65*	ABC, insecticide	NS
	*Sfl*	Est	NS

a Full detail on the genes can be found in Supplementary Table 1.

b Functional evidence associated with each gene on FlyBase (Gramates *et al.* 2022) is encoded as follows (see *Materials and Methods*): P450 = cytochrome P450; Est = carboxylesterase; ABC = ABC transporter; detox = gene involved in detoxification; insecticide = gene has a role in the response to insecticide.

c Salces-Ortiz *et al.* (2020) identified genes as being upregulated (+) or downregulated (−) in adult female gut tissue in response to malathion in each of 4 independent *D. melanogaster* strains (encoded here by letters A–D). See legend of Supplementary Table 1 for more detail.

d The reference *D. melanogaster* genome harbors *Cyp12d1-d* and *Cyp12d1-p*. These are nearly identical copies of the same gene that is subject to copy number variation in natural populations (Rivero *et al.* 2010; McDonnell *et al.* 2012; Schrider *et al.* 2013; Good *et al.* 2014; Najarro *et al.* 2015).

Interestingly, founder A5, which shows the largest increase in frequency with selection at the Chr2R QTL (Figs. 3 and 4), possesses only a single copy of *Cyp6g1*, no structural variation is evident at the gene (Chakraborty *et al.* 2019; DSPR Variant UCSC Browser 2019), and while expression of *Cyp6g1* in head tissue of adult females carrying A5 is higher than in all other DSPR A founders (bar A6), expression is lower than in A6 (see Supplementary Fig. 7 in Chakraborty *et al.* 2019). Since prior work has found that copy number and TE insertions are the major insecticide-relevant causative variation at *Cyp6g1* (Daborn *et al.* 2002; Schmidt *et al.* 2010), their absence in the A5 founder slightly reduces confidence in our assertion that variation at *Cyp6g1* underlies the Chr2R QTL. Nonetheless, other genetic factors that are less pronounced than structural variants obviously contribute to phenotype and could be present in the A5 *Cyp6g1* region. Equally, it is not implausible that the wide QTL we map are driven by variants in more than 1 gene, and a causative variant could reside in a different gene in A5 (for instance, those candidates listed in Table 2).

The cytochrome P450 gene *Cyp12d1*/FBgn0050489/FBgn0053503 is also present within the Chr2R QTL interval (Table 2). This is notable since transgenic overexpression of this gene results in greater malathion resistance (Battlay *et al.* 2018). The DSPR segregates for copy number variation at *Cyp12d1*, and of the DSPR pA founders, only founder A7 harbors 2 copies (Najarro *et al.* 2015). However, at the Chr2R QTL this founder appears to be associated with reduced resistance (Fig. 3). Coupled with the observation that *Cyp12d1* copy number is not associated with resistance toward a different insecticide, DDT (Schmidt *et al.* 2010), *Cyp12d1* seems to represent a less compelling candidate to harbor functional variation yielding the Chr2R malathion resistance QTL we map. Ultimately, our work appears to recapitulate the GWAS result showing that variation at *Cyp6g1* impacts malathion resistance (Battlay *et al.* 2018).

### A new malathion resistance locus

The malathion resistance GWAS of Battlay *et al.* (2018) revealed a set of 273 variants (of the >1.8 million tested) that survived a genomewide significance threshold [1.25 × 10^−6^—the nominal 10^−5^ threshold that is commonly used in DGRP publications (Mackay and Huang 2018) corrected for the 4 GWAS executed on different malathion phenotypes in each sex]. None of these variants are within our Chr3L QTL. One variant showed an association with a single resistance phenotype in males that survived a nominal *P *<* *10^−4^ threshold. This site is located in the *Lkr*/FBgn0035610 gene that encodes a G-protein coupled receptor involved in the regulation of feeding (Al-Anzi *et al.* 2010). Further work would be needed to evaluate whether variation at *Lkr* plays a role in malathion resistance.

Four genes at the Chr3L locus have existing annotations suggesting a role in resistance to toxicants (Table 2), 2 of which show upregulated expression in response to malathion exposure (see Salces-Ortiz *et al.* 2020). Despite the lack of an observed expression change in response to malathion, *Mdr65*/FBgn0004513 is perhaps the most likely of the candidates to segregate for insecticide-relevant variation; previous work has shown that RNAi and null mutations at the gene result in reduced malathion resistance (Sun *et al.* 2017), and CRISPR-based *Mdr65* knockouts yield lower resistance to a range of insecticides (Denecke *et al.* 2017), although malathion was not among the panel of insecticides tested. Examination of the *Mdr65* gene sequence among the DSPR pA founder chromosomes does not reveal any notable structural variation (DSPR Variant UCSC Browser 2019), although there are numerous SNPs and insertion/deletion events. Nonetheless, that our study has inferred “high” and “low” alleles at this QTL (Fig. 3) could enable explicit tests of whether *Mdr65* confers this variation, for instance by exploiting reciprocal hemizygosity testing (Steinmetz *et al.* 2002; Stern 2014) or allele swaps (Lamb *et al.* 2017). Although prior to embarking on such detailed functional work, better characterization of the effect of *Mdr65* on malathion response in the DSPR, perhaps focusing on founder alleles predicted to have different effects on phenotype, would be desirable.

### No signal of *Ace*-associated malathion resistance variation in the DSPR

Malathion is an acetylcholinesterase inhibitor. Five naturally occurring amino acid changes—F115S, I199V, G303A, F368Y, and G406A—in the *D. melanogaster Ace* gene have been shown in functional assays to confer some resistance to the toxic effects of insecticide exposure (Mutero *et al.* 1994; Menozzi *et al.* 2004; Shi *et al.* 2004). Four of these—I199V, G303A, F368Y, and G406A—segregate in the DGRP, and the first 3 are among the set of 62 variants shown in the Battlay *et al.* (2018) GWAS to survive a 3.33 × 10^−9^ threshold for at least 1 combination of malathion phenotype and sex (the minor allele for G406A is present in only 1 DGRP line and was not tested for association given its low frequency). Indeed, of these 62 hits, 8 are within the 36.6-kb of the genome spanned by the *Ace* gene, and 54 (87%) reside in a 135.4-kb genomic interval that includes *Ace*. Additionally, hits at *Ace* were identified in a GWAS for resistance to parathion (Duneau *et al.* 2018), another organophosphate insecticide.

Despite the evidence of the role of *Ace* in insecticide resistance, we do not map a QTL at the *Ace* gene in our study, and all −log_10_(*P*) values in the region are well below our genomewide statistical threshold (Fig. 2). Examining the DSPR founder sequences (King, Merkes, *et al.* 2012; Chakraborty *et al.* 2019) reveals that none of the 5 functional changes listed above are present. This is perhaps because all the DSPR founders are derived from flies collected from nature in the mid-1950s to the late 1960s (Supplementary Table 1 in King, Merkes, *et al.* 2012), right around the period when organophosphate insecticides started to be deployed widely (Casida and Quistad 1998). Thus, while we clearly lack power to find all variants associated with phenotype (Macdonald *et al.* 2022), and there will certainly be lab-to-lab variation in the resistance assay employed, since previous studies have identified massive effects on organophosphate resistance at variants in/near *Ace* (Battlay *et al.* 2018; Duneau *et al.* 2018), the absence of QTL implicating *Ace* in our study is most likely due to limited *Ace*-linked, insecticide-relevant variation present in our mapping panel.

### Differences in the inferred architecture of trait variation driven by the genetic analysis strategy employed

Similar to our previous studies with the DGRP and DSPR panels of inbred strains (Najarro *et al.* 2015, 2017; Everman *et al.* 2019), the present study again shows that genetic dissection of a given trait with different approaches can reveal a distinct underlying architecture. We appear to replicate the previously identified effect on malathion resistance of natural allelic variation at *Cyp6g1*, but fail to repeat the hit at *Ace*, most likely because our base population does not segregate for strong-effect functional variants at the gene.

We additionally identify a QTL at a position in the genome where no genomewide significant malathion resistance hits were discovered in the DGRP (Battlay *et al.* 2018). This could be because the variant(s) giving rise to this QTL are absent in the DGRP. Indeed, only a fraction of the segregating variation in the 2 panels is shared (King and Long 2017). Alternatively, the causative variant(s) may be at very low frequency in natural populations. This would render them challenging to identify using any population-based GWAS approach (Spencer *et al.* 2009), but if captured in the founders, would be amenable to discovery in a multiparental advanced intercross design like the DSPR (King, Macdonald, *et al.* 2012). Finally, the variant(s) could simply have modest effects. With just 200 inbred lines, power to identify a site explaining 4% of the genetic variation for a trait in the DGRP is below 10% (Mackay and Huang 2018), so power deficits could explain the absence of a DGRP GWAS hit within the region of our Chr3L locus. Exact reasons aside, our work adds to the evidence showing that different genetic dissection study designs can provide complementary insight into trait variation.

### An X-QTL approach is practical and efficient for genetic dissection of resistance traits

The present study did not fulfill all criteria enabling the highest resolution, highest power X-QTL study. Ideally, we would have executed selection on larger cohorts of individuals, applied stronger selection, simultaneously retained larger pools of selected animals, and repeated the experiment several more times (see Macdonald *et al.* 2022 for optimal parameters based on simulations). Nonetheless, similar to our previous study (Macdonald *et al.* 2022), we succeeded in replicating loci previously identified for our target xenobiotic resistance trait. The X-QTL approach is fairly efficient in terms of personnel time and is particularly effective for stress/toxicant resistance phenotypes; it is generally straightforward to conceive of bulked phenotyping regimes for such traits, and the experimental animals “self sort” into the selected cohort—resistant animals remain alive at some time following a challenge.

Of course, the bulked phenotyping/genotyping X-QTL approach we leverage here will not be appropriate for all laboratories, or all questions. Unlike assaying a trait in a series of stable, genotyped strains, pooled sequencing is necessary. That said, with the necessary coverage per pool on the order of 30–50× (above, and Macdonald *et al.* 2022), this can be cost-effective for *D. melanogaster* with current short-read sequencing technologies. X-QTL mapping also does not result in individual-level genotypes or phenotypes. This means one cannot take advantage of other phenotypes measured on the same genotypes to more directly explore connections among traits (the DGRP and DSPR panels have both been examined for many organismal and molecular phenotypes). It also renders X-QTL unable to dissect the contribution of epistasis to trait variation, an important phenomenon (Ehrenreich 2017) that may nonetheless generally explain only a minority of complex trait variation (Bloom *et al.* 2015; Albert *et al.* 2018; Hivert *et al.* 2021). It is possible to obtain individual-level genotypes for extremely large collections of yeast recombinants (Nguyen Ba *et al.* 2022), and such novel approaches enable genetic mapping strategies not limited by the requirement to genotype pools. However, these approaches are not easily adapted to obligate sexual *Drosophila*, as barcoded RILs would need to be individually maintained, and then pooled/assayed within a single generation.

### X-QTL dissection of insecticide resistance traits may benefit from the development of novel mapping populations

The DSPR is created from a set of strains derived from wild-caught individuals captured prior to widespread deployment of organophosphate insecticides (e.g. malathion), and well before routine use of many now commonly used insecticide classes (e.g. pyrethroids, neonicotinoids). And it appears the DSPR does not segregate for variation in at least 1 key insecticide target gene (i.e. *Ace*), variation that is known to exist in current natural populations. This suggests that a DSPR-derived base population may not be optimal for genetic dissection of all insecticide response traits via X-QTL mapping. Instead, the powerful X-QTL approach might be more profitably employed using novel, outbred, highly recombinant multiparental populations, subject to the constraints of starting from sequenced founders to enable accurate haplotype inference from pooled sequencing with modest coverage, and allowing the founders to intercross for many generations to yield fine-scale QTL mapping. Such populations would have similar properties to a DSPR-based X-QTL design (see Macdonald *et al.* 2022), but enable deeper exploration of insecticide resistance in current populations. For instance, since not all the genetic variation contributing to insecticide resistance appears to takes the form of intermediate-frequency polymorphisms of large-effect (e.g. Denecke *et al.* 2017; Schmidt *et al.* 2017), and given the modest power for low-frequency and/or small-effect variants in the DGRP (Mackay and Huang 2018), an X-QTL design could be exploited to discover genes that segregate for rare or small-effect insecticide-relevant genetic variation in contemporary populations of *D. melanogaster*.

## Data Availability

Raw malathion X-QTL sequencing data generated for this project are available on the NCBI SRA under BioProject Accession PRJNA857080, while the DSPR founder FASTQs required for analysis are available via Accession SRP011971. Scripts to run the analyses presented are available on GitHub (https://github.com/sjmac/malathion-dspr-xqtl), and other supplementary information is available on figshare (https://doi.org/10.25387/g3.21200395).
